# Deep learning-based system for automatic prediction of triple-negative breast cancer from ultrasound images

**DOI:** 10.1007/s11517-022-02728-4

**Published:** 2022-12-21

**Authors:** Alexandre Boulenger, Yanwen Luo, Chenhui Zhang, Chenyang Zhao, Yuanjing Gao, Mengsu Xiao, Qingli Zhu, Jie Tang

**Affiliations:** 1 Department of Computer Science and Technology, Tsinghua University, Haidian District, Beijing, 100084 China; 2 Department of Ultrasound, Peking Union Medical College Hospital, Chinese Academy of Medical Science & Peking Union Medical College, Shuaifuyuan 1St, Beijing, 100730 China

**Keywords:** Triple-negative breast cancer, Ultrasound, Deep learning

## Abstract

To develop a deep-learning system for the automatic identification of triple-negative breast cancer (TNBC) solely from ultrasound images. A total of 145 patients and 831 images were retrospectively enrolled at Peking Union College Hospital from April 2018 to March 2019. Ultrasound images and clinical information were collected accordingly. Molecular subtypes were determined from immunohistochemical (IHC) results. A CNN with VGG-based architecture was then used to predict TNBC. The model’s performance was evaluated using randomized k-fold stratified cross-validation. A t-SNE analysis and saliency maps were used for model visualization. TNBC was identified in 16 of 145 (11.03%) patients. One hundred fifteen (80%) patients, 15 (10%) patients, and 15 (10%) patients formed the train, validation, and test set respectively. The deep learning system exhibits good efficacy, with an AUC of 0.86 (95% CI: 0.64, 0.95), an accuracy of 85%, a sensitivity of 86%, a specificity of 86%, and an F1-score of 0.74. In addition, the internal representation features learned by the model showed clear differentiation across molecular subtype groups. Such a deep learning system can automatically predict triple-negative breast cancer preoperatively and accurately. It may help to get to more precise and comprehensive management.

**Graphical Abstract:**

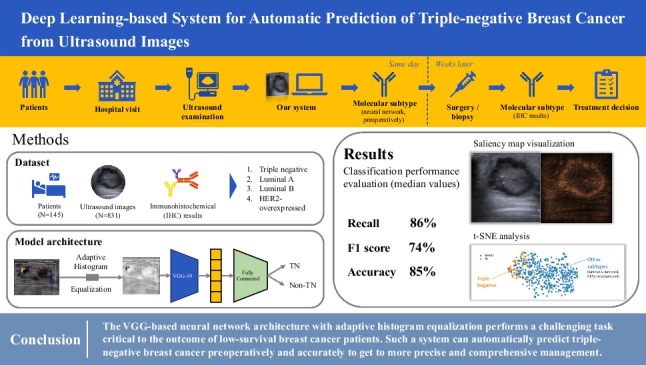

## Introduction

Breast cancer is distinguished by its high incidence and mortality rate, which puts a severe threat to women’s health worldwide [1]. The molecular subtype of breast cancer is essential to identify substantially varied clinical phenotypes, treatment responses, and outcomes [2]. Triple-negative breast cancer (TNBC) tends to be more aggressive and resistant to common treatments with a high recurrence rate and poor prognosis [3, 4]. To develop an appropriate therapy and improve the prognosis of TNBC patients, it is crucial to distinguish TNBC from the other three subtypes. In clinical practice, the molecular subtype can only be determined with certainty through surgical resection, for tumor heterogeneity can lead to the existence of multiple molecular types in a tumor, and the samples captured by core biopsy only constitute a small portion of the whole lesion, not always representative of heterogeneous tumors. Additionally, for advanced patients, neoadjuvant therapy plays an essential role in the treatment plan. The molecular subtype of a breast lesion may change after treatment [5]. More importantly, it has been shown that the biological characteristics of residual lesions after neoadjuvant therapy have a greater impact on the prognosis, rather than the characteristics of the primary tumor [6]. And the conversion to triple-negative after neoadjuvant therapy is an independent risk factor affecting the prognosis [5]. In this case, another biopsy is required for further therapies. It is of great value to develop a noninvasive, accurate, and efficient approach to determine the molecular subtype of breast cancer.

Medical imaging plays an essential part in the assessment of breast cancer, as the primary tool to detect and diagnose lesions. Nevertheless, the role of imaging is undergoing a rapid evolution from merely providing diagnostic information to leading the advancement of personalized precision medicine, with the permeation of deep learning into the field of medical imaging [7]. Deep learning (DL) has exhibited promising performance on a range of diagnostic and predictable tasks on medical images [8–10]. A multicenter study has achieved satisfying performance (with an AUC of 0.91) to differentiate three breast cancer molecular subtypes on MRI using deep learning algorithms [11]. Another study developed a deep learning mammography-based model that identified women at high risk of breast cancer [8]. These advances motivate the use of deep learning for molecular subtype determination from medical images.

Ultrasound (US) is a common imaging modality that uses sound waves to produce images of body structures [12, 13], including breast, thyroid, muscles, joints, vessels, and internal organs. The images can provide valuable information for diagnosing and directing treatment for diseases. It is a preferred medical imaging method for breast cancer and has the highest adoption rate in Asian countries, for its noninvasiveness, convenience, and high sensitivity to breast nodules in the dense breast [14, 15]. However, the acquisition of US images is prone to discrepancies between operators. Artifacts during the US image acquisition, like noise, speckle, and signal attenuation, can make it difficult for radiologists to identify the disease. More importantly, because of variability in equipment and grayscale adjustments, the size, format, and grayscale of the captured images also vary. There is no standardized method to acquire images, and this poses challenges for applications using US images. Only a few deep learning studies use breast ultrasound as a modality, compared to mammography and MRI, and most of them focus on the development of deep learning approaches to assist the detection, segmentation, and diagnosis of breast cancer [16–19]. Few studies have explored distinguishing molecular subtypes solely from raw ultrasound images, and the performance varies widely across molecular subtypes [20–23].

In this study, we aimed to develop a fully automated deep learning-based system for molecular subtype solely from breast cancer ultrasound images and evaluate the ability of the system to distinguish TNBC, which has the poorest diagnosis and prognosis, from other cases (luminal A, luminal B, or HER2-positive).

## Materials and methods

This retrospective study was approved by the Institutional Review Board of Perking Union Medical College Hospital (Number: JS-1987), and written informed consent was obtained from all the participants.

### Study cohorts and datasets

**Fig. 1:**
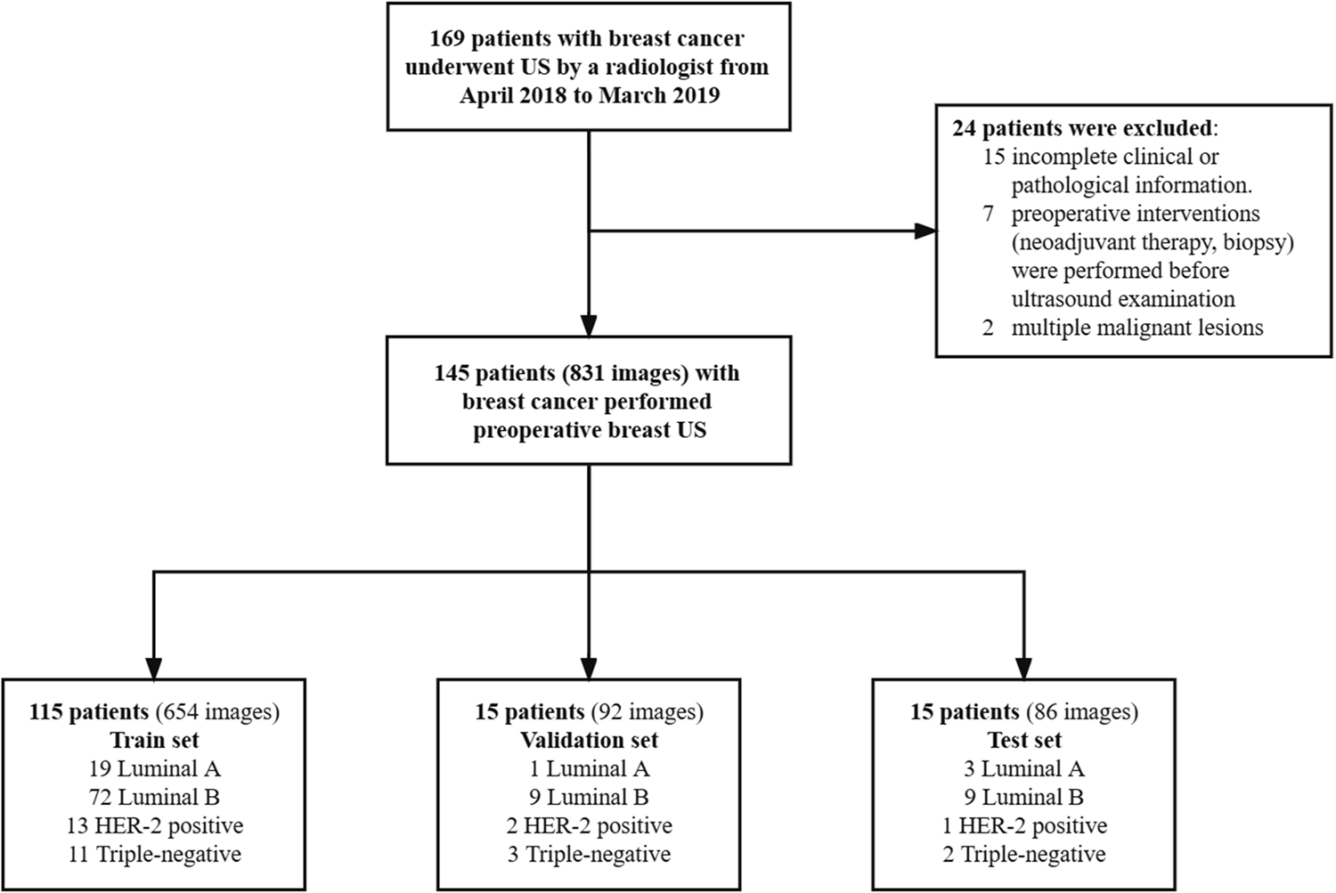
Flowchart of procedure in this study

The dataset was collected at Peking Union Medical College Hospital and consists of 145 female breast cancer patients without a breast cancer history who underwent ultrasound examination by a single radiologist between April 2018 and March 2019. Exclusion criteria were the following: (1) preoperative intervention (neoadjuvant therapy (NAT), biopsy) performed before ultrasound examination. Biopsy affects the tumor morphology by cutting a part of the tumor. NAT results in changes in tumor size, morphology, and even clone. These changes do not reflect the true condition of the tumor. (2) multiple malignant lesions. Patients with multiple breast cancers have significantly poorer disease-free survival than those with a single tumor [24]. The tumor multiplicity is used as an independent factor for subclassifying breast cancer. So, multiple breast cancer cases were excluded from this study. (3) incomplete clinical or pathological information. The patients were divided into train, validation, and test sets randomly at a ratio of 8:1:1. A flowchart summarizing these steps is shown in Fig. 1.

For each patient who underwent US examination, two or more US images were captured using ACUSON S2000 (Siemens) and EPIQ7 (Philips) machines with linear probes (3–12 MHz) by an experienced radiologist (with 11 years of experience) and reviewed by two experienced radiologists (with 6 and 4 years of experience, respectively) to confirm the index lesion. The index tumor images were captured from multiple angles, including at least longitudinal and transversal sections. US tumor size was measured at the longitudinal section. Both grayscale and color Doppler images were included.

### Subtype labeling

The St. Gallen International Breast Conference proposed estrogen receptor (ER), progesterone receptor (PR), human epidermal growth factor receptor type 2 (HER2) protein, and the Ki-67 proliferation index as the main receptor indicators for the molecular subtype of breast cancer [25]. The above factors are strongly associated with the prognosis and outcome of breast cancer patients [26–30]. According to this, each patient underwent a surgical excision to obtain the tumor’s biological marker status evaluation via immunohistochemical (IHC) staining, namely the estrogen receptor (ER), the progesterone receptor (PR), the human epidermal growth factor receptor type 2 (HER2) protein, and the Ki-67 proliferation index.

The molecular subtype was labeled using the standard criteria applied to biological marker status evaluation referring to the surrogate definition of intrinsic molecular subtypes of breast cancers from the St. Gallen International Breast Cancer Conference [25]: luminal A (ER (+) or PR (+), Ki-67 low (< 14%) and HER2 (−), luminal B (ER (+) or PR (+), Ki-67 high (≥ 14%), and/or HER2 (+)), HER2-positive (ER (−), PR (−) and HER2 (+)), and triple-negative (ER (−), PR (−), HER2 (−)).

Note, ER or PR is considered positive when the percentage of stained cells is > 1%. For HER2, a score of 0 or 1+ is considered negative, and a score of 3+ is considered positive. If IHC scored 2+, FISH is further tested, and HER2 is considered positive if the ratio of the HER2 gene signal to the chromosome 17 probe signal (HER2/CEP17 ratio) is ≥ 2.0, or the average HER2 signals/cell is ≥ 6.0.

Given the poorer prognosis of triple-negative cases, we grouped the non-triple-negative (luminal A, luminal B, and HER2-positive) cases and focused on the binary classification task of identifying triple-negative cases.

### Image preprocessing

**Fig. 2:**
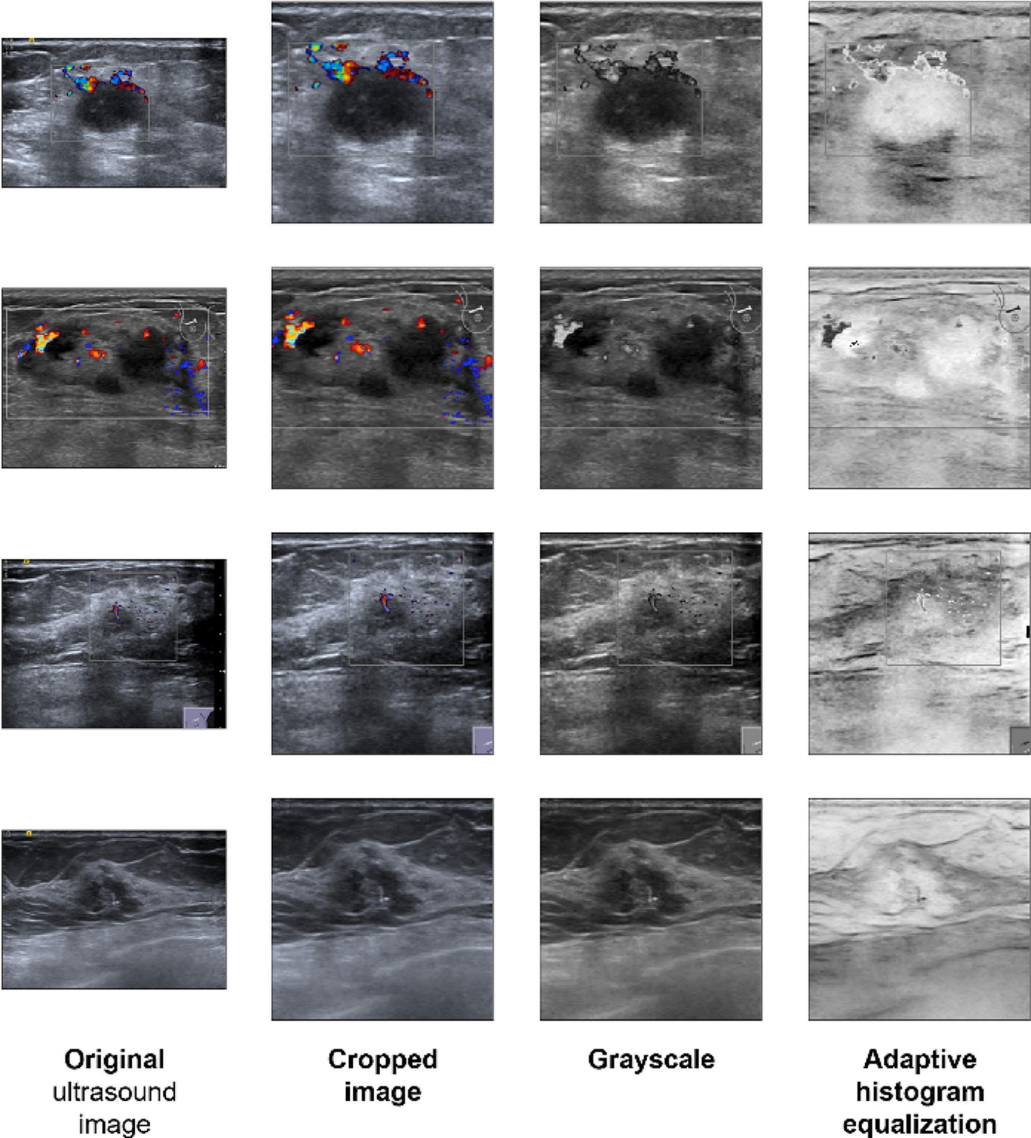
Image processing steps. Images are resized and cropped to 224 × 244 pixels, converted to grayscale, and then altered by adaptive histogram equalization

First, images were resized and cropped to have a uniform model input size and also mitigate the presence of noise (e.g., black bands) in the outer parts of the images. Then, we preprocessed the US images to deal with the problem of intensity heterogeneity in ultrasound, whereby a tumor tissue of the same nature (e.g., a cancerous tissue) appears with varying pixel intensity across images, depending on the settings used by the ultrasound machine operator. It is a common problem and a major challenge in automated inference from ultrasound images. We transformed the images using adaptive histogram equalization [31]. This contrast enhancement method adjusts the intensity of pixels across an image to normalize the local histograms of pixel values (Fig. 2). It computes multiple histograms, one for each section of the image, and uses them to balance the intensity values of the image. We assessed the effectiveness of several other approaches to counter the intensity heterogeneity, including standard dataset-level normalization (mean centering and standard scaling) or image-level normalization, and found the adaptive histogram equalization algorithm to be the most effective.

### Model architecture

**Fig. 3:**
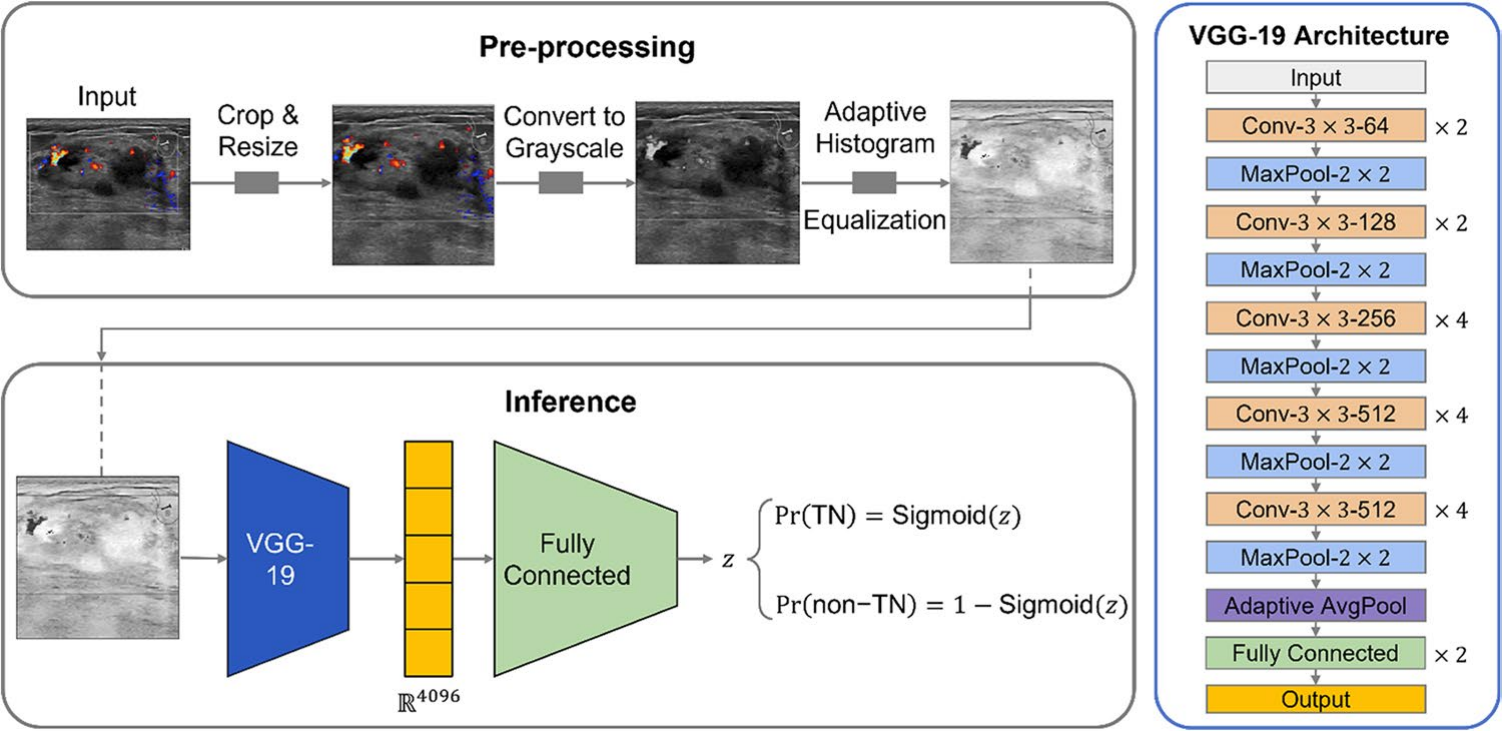
End-to-end system and architecture of the model. VGG-19 architecture modified to perform binary classification, preceded by adaptive histogram equalization

For automatic classification, a VGG-based model was employed to distinguish triple-negative tumors from other tumors using pixel information presented in US images, given the popularity and success of the VGG model in the medical field [32]. The model uses solely US images as input, without a delineated region of interest (ROI), to predict the molecular subtype of a patient. Fig. 3 shows the components of the system and the architecture of the model.

We alter the VGG-19 architecture [33] to perform binary classification of raw ultrasound images into the triple-negative class or the rest class, end-to-end. For each patient, each image is classified independently from the patient’s other images. This allows us to assess the model’s generalization across the different angles of capture and variations in the grayscale or color Doppler ultrasound. For this, we ensure all the images of a patient appear in one and only one of the different image sets used while training the model.

### Model training

As opposed to most other studies that employ such models in the medical field, we do not pre-train the model on a larger, unrelated dataset, but instead, we train it from scratch. We do so since all available pre-trained models are trained on datasets of natural images such as ImageNet, where the pixel distribution differs fundamentally from those of ultrasound images.

To tune the model, we adopt a train, validation, and test setup. The model is trained to learn visual patterns using the cross-entropy loss with standard backpropagation [34, 35]. The model is trained to learn image patterns for each of the classes on the train set (80%), and the validation (10%) and test sets (10%) are left out of the sample, i.e., not seen during training. Here, we report the out-of-sample performance as measured on the test set. Note, the data is partitioned in the space of patients and not in the space of images so that distinct images of a patient all appear in one and only one of these three sets.

### Performance evaluation

The model’s performance was measured using k-fold stratified cross-validation. Each fold is a random partition of the dataset, and at each fold, the proportion of each of the two classes in the three subsets is the same as those in the overall dataset. To further counter the class imbalance, we under-sample the dominant class (rest) at each epoch.

**Fig. 4:**
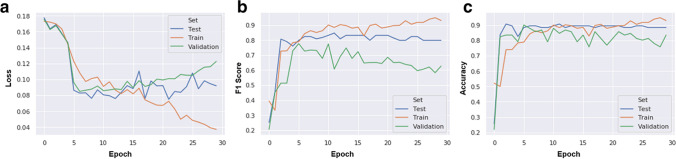
Performance of the model. Loss (summation of errors in the model) (**a**), F1-score (harmonic mean of the model’s precision and recall) (**b**), and accuracy (proportion of classifications a model made correctly) (**c**) of the model at each epoch on the train, validation, and test sets of one of the four partitions

We use Adam as a learning rate schedule [36], but find that manually reducing the learning rate helps gain a few percentage points in the performance. We cap the number of training epochs to 30 and apply an early-stopping scheme. The early stopping epoch and hyper-parameters are set according to the performance of the validation set. The loss curves and evolution of the model’s classification performance through training epochs (learning curves) are shown in Fig. 4.

To exhibit the performance of the model, the area under the receiver operating characteristic curve (AUC) with a 95% confidence interval (CI) is reported. The accuracy, specificity, sensitivity values, and F1-score with 95% CIs are reported. In addition, the number of true-positive, false-positive, true-negative, and false-negative findings of the model are reported in a confusion matrix. These statistical analyses were performed using SPSS software (version 25.0, IBM, Armonk, NY, USA).

To show the interpretability of the model, t-SNE (t-distributed Stochastic Neighbor Embedding) analysis and saliency maps were used to visualize the features learned by the model and the areas of the images that are most suggestive of triple-negative breast cancer in the model.

## Results

### Patients

A total of 145 patients (mean age, 51.66±11.13 years; range, 29–82 years) were enrolled in this study. Baseline information on the study population is detailed in Table 1. The dataset was partitioned into train, validation, and test cohorts, with, for example, in the case of Partition 1, 115 (79.31%), 15 (10.34%), and 15 (10.34%) breast cancer patients respectively. There was no significant difference among the three cohorts with respect to age (*P* = 0.667), US tumor size (*P* = 0.178), histological type distribution (*P* = 0.351), and molecular subtype (*P* = 0.840). Molecular subtypes have an incidence in the dataset in proportions comparable with the incidence in the broader population [37].

**Table 1:** Baseline information for the train, validation, and test sets

Characteristics	Train	Validation	Test	*P*-value
Population size	115 (79.32%)	15 (10.34%)	15 (10.34%)	-
Age (mean ± SD, years)	52.04 ± 11.42	49.40 ± 10.15	50.93 ± 10.07	0.667
US tumor size (mean ± SD, cm)	2.62 ± 1.29	2.86 ± 2.52	1.95 ± 1.14	0.178
Histological type				0.351
Invasive ductal carcinoma (IDC)	87 (75.65%)	9 (60.00%)	9 (60.00%)	-
Invasive lobular carcinoma (ILC)	5 (4.35%)	1 (6.67%)	0 (0.00%)	-
Ductal carcinoma in situ (DCIS)	14 (12.17%)	4 (26.66%)	5 (33.33%)	-
Other types	9 (7.83%)	1 (6.67%)	1 (6.67%)	-
Molecular subtype				0.840
Luminal A	19 (16.52%)	1 (6.67%)	3 (20.00%)	-
Luminal B	72 (62.61%)	9 (60.00%)	9 (60.00%)	-
HER2-positive	13 (11.30%)	2 (13.33%)	1 (6.67%)	
Triple-negative	11 (9.57%)	3 (20.00%)	2 (13.33%)	

### Model performance

The effectiveness of the model at distinguishing triple-negative tumors from the other three molecular subtypes was assessed using four-fold cross-validation. We report in Table 2 the median of metrics across the four partitions. The model reaches an AUC of 0.86 (95% CI: 0.64, 0.95), a sensitivity of 86%, a specificity of 86%, and an F1-score of 0.74 on the test set.

**Table 2:** Classification performance on the test set across the four partitions

Partition	AUC	Sensitivity	Specificity	PPV	NPV	F1-score
1	0.8155 (0.6813, 0.9429)	0.7143 (0.4667, 0.9333)	0.9167 (0.8472, 0.9722)	0.6250 (0.3750, 0.8571)	0.9429 (0.8873, 0.9863)	0.7981 (0.6729, 0.9016)
2	0.9067 (0.8600, 0.9494)	1.0000 (1.0000, 1.0000)	0.8133 (0.7260, 0.8987)	0.3636 (0.1739, 0.5714)	1.0000 (1.0000, 1.0000)	0.7152 (0.5798, 0.8362)
3	0.7899 (0.6416, 0.9293)	0.6667 (0.3846, 0.9286)	0.9130 (0.8438, 0.9726)	0.5714 (0.3000, 0.8235)	0.9403 (0.8788, 0.9857)	0.7709 (0.6318, 0.8889)
4	0.9016 (0.8500, 0.9508)	1.0000 (1.0000, 1.0000)	0.8033 (0.6964, 0.9000)	0.3684 (0.1500, 0.6000)	1.0000 (1.0000, 1.0000)	0.7147 (0.5662, 0.8383)
*x*˜	**0.8586**	**0.8571**	**0.8632**	**0.4699**	**0.9714**	**0.7431**

Data in parentheses are 95% confidence intervals

*PPV* positive predictive value, *NPV* negative predictive value, *AUC* area under the receiver operating characteristic curve

**Fig. 5:**
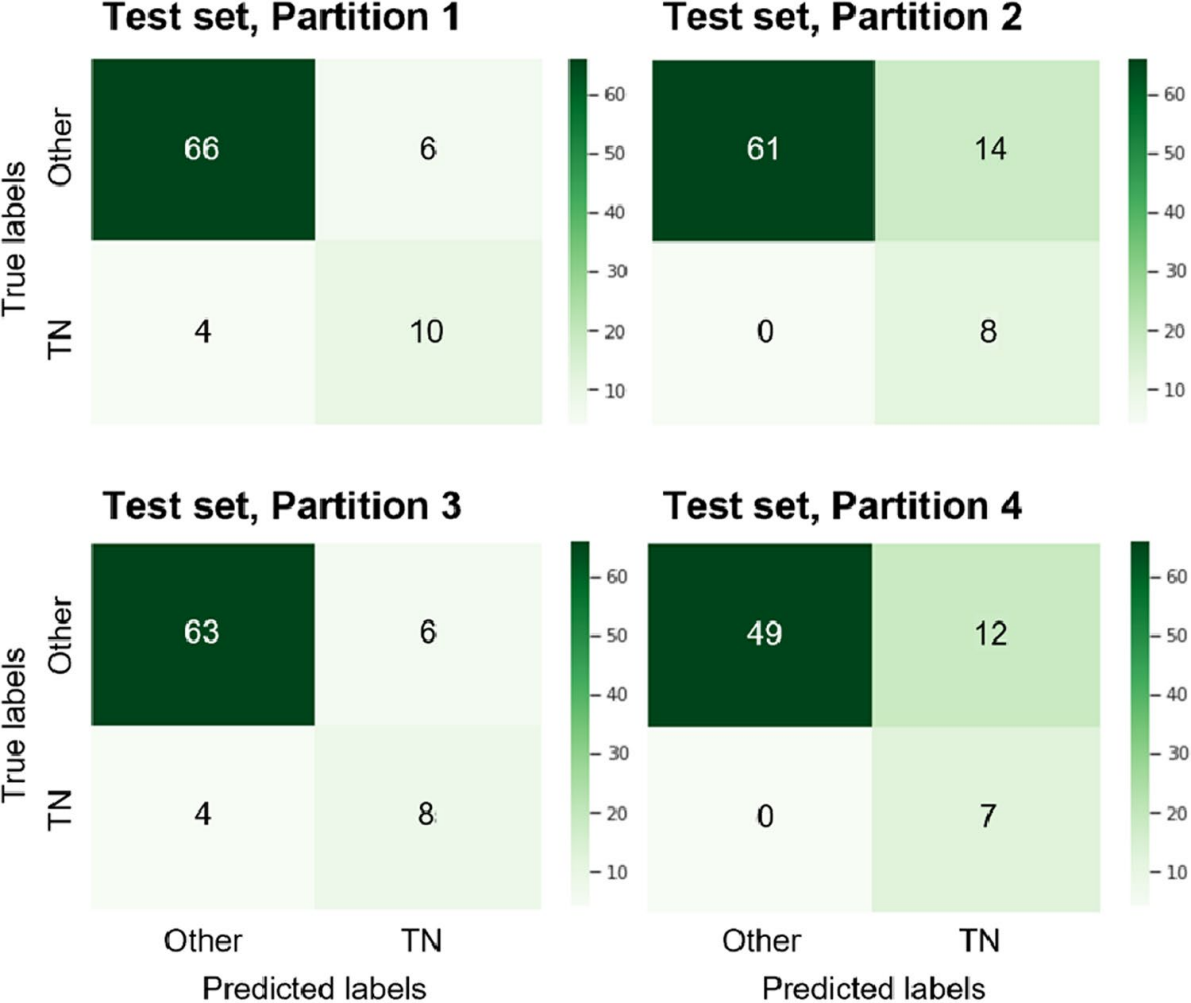
Confusion matrix of the classification results. Rows are the true class (ground truth) of test examples and columns are the predicted class

The metrics are best understood via the confusion matrices in Fig. 5, which show the exact number of correct and incorrect classifications for each class and each dataset partition.

### Feature visualization

**Fig. 6:**
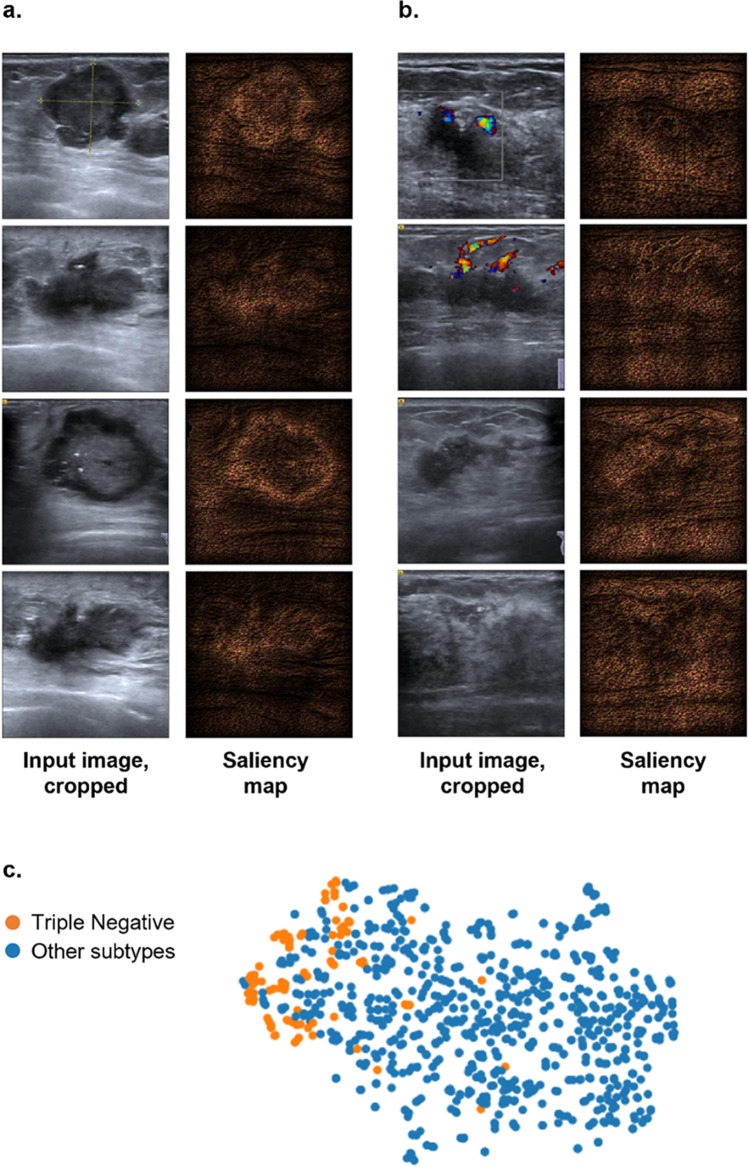
Visualization of the model’s internal features. **a**–**b** Cropped original images and saliency maps of four triple-negative patients (**a**) and four non-triple-negative patients (**b**), where highlighted pixels are those with greater influence on the model’s classification decision. **c** t-SNE visualization of convolutional features in the last hidden layer of the trained model, exhibiting class separation in internal high-level feature representations

We visualize in Fig. 6 the internal features learned by the model using t-SNE. Each color corresponds to a class from the dataset, and each dot represents a breast ultrasound image from the dataset, projected from the 4096-dimensional output of the model’s last hidden layer into two dimensions. Two clusters of dots can be identified, exhibiting class separation. The triple-negative cluster lies on the edge of the cloud of dots, highlighting that triple-negative cases are visually distinguishable, to the extent that the model learns a high-level representation in which triple-negative cases are separable from other cases (luminal A, luminal B, HER2-positive).

Furthermore, we produce saliency maps to understand the visual features of breast tumors, as seen on the ultrasound images, used by the model to classify images (Fig. 6). Saliency maps highlight the pixels of an input image that most influence the model’s classification decision, for images randomly sampled from the dataset for each of the two classes. The maps are computed by taking each pixel’s gradient with respect to the model’s loss function. On a saliency map, highlighted pixels are those with greater influence on the model’s classification decision. This shows that the CNN network focuses on the most predictive part of the image. TN saliency maps display higher brightness on hypoechoic lesions of TN, suggesting that the model relies more heavily on information from the tumor tissue and margin in TN cases. In contrast, for saliency maps of other types (non-TN), brightness is more uniform across both lesion and background areas. This indicates the model indifferently uses information from the lesion or neighboring tissue.

## Discussion

Triple-negative breast cancer often occurs at a young age and presents the highest degree of malignancy and invasiveness. Unfortunately, endocrine therapy and targeted therapy cannot benefit patients, who may only rely on chemotherapy [2, 4, 38]. Therefore, identifying triple-negative breast cancer is the key to guiding the selection of clinical pathways.

In this study, we present a model that automatically distinguishes triple-negative breast cancer from other molecular subtypes in a non-invasive, comprehensive manner. The model is trained on US images, without any histopathological information as predictive input, and achieves an area under the receiver operating characteristic curve (AUC) of 0.86, a sensitivity of 85.7%, and a specificity of 86.3%, promising for the task of predicting the biological behavior of TNBC preoperatively. Our proposed approach demonstrates the potential of CNN models to automatically identify triple-negative patients based on US images preoperatively and can assist in making more appropriate treatment decisions.

Although distinguishing breast cancer molecular subtypes from US images is a relatively new research field, previous studies have found differences between TN and non-TN tumors in visual US features; TN tumors are more likely to have a circumscribed margin but less likely to present calcifications and echogenic halo [39–43]. Previously, ultrasonic features of invasive breast ductal carcinoma were extracted and selected using machine learning methods, and those features demonstrated a strong correlation with receptor status and molecular subtypes [44]. Also, it has been reported that some features mined from ultrasonic imaging could distinguish TNBC and fibroma [45]. Recently, the use of deep learning has helped advance the automated classification of molecular subtypes of breast cancer. In the existing literature, only four other studies tackle the determination of breast cancer molecular subtypes solely from raw ultrasound images. A study developed three deep learning models that determine the molecular subtype from multi-modal US images, including a monomodal model (grayscale US), a dual-modal model (grayscale US and color Doppler), and a multimodal model (grayscale US, color Doppler, and shear-wave elastography, SWE) [22]. Two other studies first performed benign-malignant identification and later inferred the molecular subtype separately [21, 23], while another study put more emphasis on the task of discriminating luminal and non-luminal cases [20]. However, the predictive ability for triple-negative breast cancer varied widely across studies (accuracy range of 53.19–97.02%) and could be further improved. In contrast with previous studies, we conducted a discriminative prediction of TN and non-TN. We employed VGG-19, a convolutional neural network architecture different from previous studies (ResNet50 and Xception for the first two [36] and for the third one [6], respectively). Our model achieves superior performance when considering grayscale US and color Doppler, with an AUC of 0.86 and an F1-score of 0.74. Also, we used a method for standardization of ultrasound images, in which images are preprocessed to eliminate the effect caused by intensity variability—beneficial to the model’s generalization ability.

After observing this attractive performance, to understand how the model learned from the input data to discriminate TN from others, two analytical methods were employed to visualize the model’s learned internal features. Feature visualization is needed in part to confirm the model indeed focuses on US features associated with triple-negative cases rather than irrelevant parts of the image. First, t-SNE analysis shows that in the learned feature space, TN and other cases are separable. Second, the saliency maps produced are an intuitive reflection of the different weights given by the model to visual features in US images. For TN lesions, the model gives greater importance to pixel information from the tumor tissue and margin, as seen by the higher brightness of hypoechoic lesions on TN saliency maps. However, for lesions of other types, the model indifferently uses information from the lesion or neighboring tissue, as seen by the more uniform brightness across both lesion and background areas on saliency maps of other subtypes (non-TN). This is consistent with previous findings that under grayscale ultrasound, triple-negative lesions tend to have more circumscribed margins and can be clearly distinguished from surrounding tissue while non-TN lesions are typically less differentiated from surrounding tissue and have lower contrast and more irregular shape [39, 40].

Our study presents several limitations. First, the sample is small, and the data were collected from a single center, so the predictive ability of the model needs to be validated on further external data in a multi-center setup—a necessary step toward clinical use. Second, in this study, we collected US images presenting at least the largest diameter sections and the orthogonal section. It remains debatable whether the predictive ability of the model would be significantly affected by including additional US images from a single index lesion. Third, while our study focuses on the binary problem of identifying triple-negative cases, future research should tackle the four-way breast cancer molecular subtyping task. Fourth, benchmarking several deep learning models (including non-CNN ones, e.g., Vision Transformers [46]) would help identify the architecture best suited for the task at hand and achieve performance gains.

## Conclusion

An end-to-end deep learning approach was proposed to identify in raw ultrasound images triple-negative breast cancer, characterized by its poor diagnosis and prognosis—a task that radiologists are not able to perform. The approach is non-invasive and automated, as it does not use any histopathological information from biopsy or surgery as predictive input and does not rely on manually crafted features like region of interest or radiomics. The system can serve as a prospective decision-making tool for clinicians enacting treatment plans and assessing prognosis.

## Abbreviations

TNBC: Triple-negative breast cancer
DL: Deep learning
US: Ultrasound
IHC: Immunohistochemical
ER: Estrogen receptor
PR: Progesterone receptor
HER2: Human epidermal growth factor receptor type 2

## References

[1] Siegel RL, Miller KD, Fuchs HE, Jemal A (2021). Cancer statistics, 2021. CA Cancer J Clin.

[2] Carey LA, Perou CM, Livasy CA, Dressler LG, Cowan D, Conway K (2006). Race, breast cancer subtypes, and survival in the Carolina Breast Cancer Study. JAMA.

[3] Liedtke C, Mazouni C, Hess KR, André F, Tordai A, Mejia JA (2008). Response to neoadjuvant therapy and long-term survival in patients with triple-negative breast cancer. J Clin Oncol.

[4] Kennecke H, Yerushalmi R, Woods R, Cheang MC, Voduc D, Speers CH (2010). Metastatic behavior of breast cancer subtypes. J Clin Oncol.

[5] Li C, Fan H, Xiang Q, Xu L, Zhang Z, Liu Q (2019). Prognostic value of receptor status conversion following neoadjuvant chemotherapy in breast cancer patients: a systematic review and meta-analysis. Breast Cancer Res Treat.

[6] Tacca O, Penault-Llorca F, Abrial C, Mouret-Reynier MA, Raoelfils I, Durando X (2007). Changes in and prognostic value of hormone receptor status in a series of operable breast cancer patients treated with neoadjuvant chemotherapy. Oncologist.

[7] Esteva A, Robicquet A, Ramsundar B, Kuleshov V, DePristo M, Chou K (2019). A guide to deep learning in healthcare. Nat Med.

[8] Yala A, Lehman C, Schuster T, Portnoi T, Barzilay R (2019). A deep learning mammography-based model for improved breast cancer risk prediction. Radiology.

[9] McKinney SM, Sieniek M, Godbole V, Godwin J, Antropova N, Ashrafian H (2020). International evaluation of an AI system for breast cancer screening. Nature.

[10] Aggarwal R, Sounderajah V, Martin G, Ting DSW, Karthikesalingam A, King D (2021). Diagnostic accuracy of deep learning in medical imaging: a systematic review and meta-analysis. NPJ Digit Med.

[11] Zhang Y, Chen JH, Lin Y, Chan S, Zhou J, Chow D (2021). Prediction of breast cancer molecular subtypes on DCE-MRI using convolutional neural network with transfer learning between two centers. Eur Radiol.

[12] Leighton TG (2007). What is ultrasound?. Prog Biophys Mol Biol.

[13] Ensminger D, Bond L (2011) Ultrasonics: fundamentals, technologies, and applications, third edition.

[14] Brem RF, Lenihan MJ, Lieberman J, Torrente J (2015). Screening breast ultrasound: past, present, and future. AJR Am J Roentgenol.

[15] Park YH, Senkus-Konefka E, Im SA, Pentheroudakis G, Saji S, Gupta S (2020). Pan-Asian adapted ESMO Clinical Practice Guidelines for the management of patients with early breast cancer: a KSMO-ESMO initiative endorsed by CSCO, ISMPO, JSMO, MOS, SSO and TOS. Ann Oncol.

[16] Fujioka T, Mori M, Kubota K, Oyama J, Yamaga E, Yashima Y et al (2020) The utility of deep learning in breast ultrasonic imaging: a review. Diagnostics (Basel). 10 10.3390/diagnostics10121055 PMC7762151 33291266

[17] Sun Q, Lin X, Zhao Y, Li L, Yan K, Liang D (2020). Deep learning vs. radiomics for predicting axillary lymph node metastasis of breast cancer using ultrasound images: don’t forget the peritumoral region. Front Oncol.

[18] Zheng X, Yao Z, Huang Y, Yu Y, Wang Y, Liu Y (2020). Deep learning radiomics can predict axillary lymph node status in early-stage breast cancer. Nat Commun.

[19] Chen C, Wang Y, Niu J, Liu X, Li Q, Gong X (2021). Domain knowledge powered deep learning for breast cancer diagnosis based on contrast-enhanced ultrasound videos. IEEE Trans Med Imaging.

[20] Jiang M, Zhang D, Tang SC, Luo XM, Chuan ZR, Lv WZ (2021). Deep learning with convolutional neural network in the assessment of breast cancer molecular subtypes based on US images: a multicenter retrospective study. Eur Radiol.

[21] Ye H, Hang J, Zhang M, Chen X, Ye X, Chen J (2021). Automatic identification of triple negative breast cancer in ultrasonography using a deep convolutional neural network. Sci Rep.

[22] Zhou BY, Wang LF, Yin HH, Wu TF, Ren TT, Peng C (2021). Decoding the molecular subtypes of breast cancer seen on multimodal ultrasound images using an assembled convolutional neural network model: a prospective and multicentre study. EBioMedicine.

[23] Zhang X, Li H, Wang C, Cheng W, Zhu Y, Li D (2021). Evaluating the accuracy of breast cancer and molecular subtype diagnosis by ultrasound image deep learning model. Front Oncol.

[24] Chu J, Bae H, Seo Y, Cho SY, Kim SH, Cho EY (2018). The prognostic impact of synchronous ipsilateral multiple breast cancer: survival outcomes according to the Eighth American Joint Committee on Cancer Staging and Molecular Subtype. J Pathol Transl Med.

[25] Goldhirsch A, Wood WC, Coates AS, Gelber RD, Thürlimann B, Senn HJ (2011). Strategies for subtypes–dealing with the diversity of breast cancer: highlights of the St. Gallen International Expert Consensus on the Primary Therapy of Early Breast Cancer 2011. Ann Oncol.

[26] Giulianelli S, Lamb CA, Lanari C (2021). Progesterone receptors in normal breast development and breast cancer. Essays Biochem.

[27] Yerushalmi R, Woods R, Ravdin PM, Hayes MM, Gelmon KA (2010). Ki67 in breast cancer: prognostic and predictive potential. Lancet Oncol.

[28] Thomas C, Gustafsson J (2011). The different roles of ER subtypes in cancer biology and therapy. Nat Rev Cancer.

[29] Britschgi A, Duss S, Kim S, Couto JP, Brinkhaus H, Koren S (2017). The Hippo kinases LATS1 and 2 control human breast cell fate via crosstalk with ERα. Nature.

[30] Marchiò C, Annaratone L, Marques A, Casorzo L, Berrino E, Sapino A (2021). Evolving concepts in HER2 evaluation in breast cancer: Heterogeneity, HER2-low carcinomas and beyond. Semin Cancer Biol.

[31] Maaten.Lvd HG (2008). Visualizing data using t-SNE. J Mach Learn Res.

[32] Litjens G, Kooi T, Bejnordi BE, Setio AAA, Ciompi F, Ghafoorian M (2017). A survey on deep learning in medical image analysis. Med Image Anal.

[33] Simonyan KZA (2015) Very deep convolutional networks for large-scale image recognition. 3rd International Conference on Learning Representations, {ICLR} 2015, San Diego, CA, USA, May 7–9Conference Track Proceedings

[34] Yu J, Zhu C, Zhang J, Huang Q, Tao D (2020). Spatial pyramid-enhanced NetVLAD with weighted triplet loss for place recognition. IEEE Trans Neural Netw Learn Syst.

[35] Yang L, He Z, Cao Y, Fan D (2022) A progressive subnetwork searching framework for dynamic inference. IEEE Trans Neural Netw Learn Syst 10.1109/TNNLS.2022.3199703 36063528

[36] He KZX, Ren S, Sun J (2016) Deep residual learning for image recognition. 2016 IEEE Conference on Computer Vision and Pattern Recognition (CVPR). 770–778

[37] Cho N (2016). Molecular subtypes and imaging phenotypes of breast cancer. Ultrasonography.

[38] Prat A, Pineda E, Adamo B, Galván P, Fernández A, Gaba L (2015). Clinical implications of the intrinsic molecular subtypes of breast cancer. Breast.

[39] Ko ES, Lee BH, Kim HA, Noh WC, Kim MS, Lee SA (2010). Triple-negative breast cancer: correlation between imaging and pathological findings. Eur Radiol.

[40] Wojcinski S, Soliman AA, Schmidt J, Makowski L, Degenhardt F, Hillemanns P (2012). Sonographic features of triple-negative and non-triple-negative breast cancer. J Ultrasound Med.

[41] Çelebi F, Pilancı KN, Ordu Ç, Ağacayak F, Alço G, İlgün S (2015). The role of ultrasonographic findings to predict molecular subtype, histologic grade, and hormone receptor status of breast cancer. Diagn Interv Radiol.

[42] Zhang L, Li J, Xiao Y, Cui H, Du G, Wang Y (2015). Identifying ultrasound and clinical features of breast cancer molecular subtypes by ensemble decision. Sci Rep.

[43] Wu T, Li J, Wang D, Leng X, Zhang L, Li Z (2019). Identification of a correlation between the sonographic appearance and molecular subtype of invasive breast cancer: a review of 311 cases. Clin Imaging.

[44] Guo Y, Hu Y, Qiao M, Wang Y, Yu J, Li J (2018). Radiomics analysis on ultrasound for prediction of biologic behavior in breast invasive ductal carcinoma. Clin Breast Cancer.

[45] Lee SE, Han K, Kwak JY, Lee E, Kim EK (2018). Radiomics of US texture features in differential diagnosis between triple-negative breast cancer and fibroadenoma. Sci Rep.

[46] Alexey Dosovitskiy LB, Alexander Kolesnikov, Dirk Weissenborn, Xiaohua Zhai, Thomas Unterthiner, Mostafa Dehghani, Matthias Minderer, Georg Heigold, Sylvain Gelly, Jakob Uszkoreit, Neil Houlsby (2020) An image is worth 16x16 words: transformers for image recognition at scale. CoRR. abs/2010.11929

